# Molecular docking analysis of beta-lactamase from Salmonella species with eicosane

**DOI:** 10.6026/97320630018669

**Published:** 2022-08-31

**Authors:** V Nagasinduja, S Shahitha, B Prakash, D Jegadeesh Kumar

**Affiliations:** 1 Department of Microbiology, Muthayammal College of Arts & Science, Namakkal, Tamil Nadu, India; 2 Department of Biotechnology, Vels Institute of Science Technology & Advanced Studies, Chennai, Tamil Nadu, India; 3 Chromopark Research Centre, Namakkal, Tamil Nadu, India

**Keywords:** eicosane, beta-lactamase, metronidazole, drug docking

## Abstract

beta-lactamases of Salmonella Sp. belongs to a group of enzymes produced by bacteria which break the beta-lactam ring to inactivate the beta-lactam antibiotic. Therefore, it is of interest to document the molecular docking analysis of beta-lactamase from Salmonella species with eicosane. Hence, we document the molecular docking analysis data of beta-lactamase from Salmonella species with eicosane.

## Background:

Several serovars of Salmonella enterica subspecies enterica cause salmonellosis in humans. Food is a large global reservoir of Salmonella. Salmonella is the 2nd top known bacteria that cause human gastero intestinal outbreaks especially with the species of Salmonella enteritidis and Salmonella typhimurium. Salmonella is the primary cause of infection in about half of the 1500 cumulative food borne infections that occurs in France every year. Salmonellosis is caused by non-typhoid Salmonella resulting in acute gastroenteritis. It is seen in 95% of cases by the intake of contaminated food specifically fresh fruit juices, meet and egg. It is also present in fresh products like fruits and vegetables which are contaminated by animal faeces [1]. The rising frequency of enterobacterial strains producing extended-spectrum lactamases is linked to ESBLs. Third-generation cephalosporins, penicillins, and monobactams are inactivated by these enzymes [2-3]. ESBL development in bacteria that aren't generally known to display lactam resistance can provide useful information about resistance gene transfer and the significance of antimicrobial control methods in animal feed [3- 4]. Even in the absence of selective pressure from antimicrobial drugs, the prevalence of ESBL carriage is likely to rise and spread to different enteric pathogens, as it did with ampicillin resistance [5] and, more recently, cephalosporin resistance in Escherichia coli [6]. All penicillin, cephalosporin, and mono lactam drugs are resistant to ESBL-producers [7]. ESBLs have developed plasmid-encoded enzyme families (TEM, SHV, cefotaxime (CTXM), and oxacillin (OXA), but they can also be encoded on the chromosome or be transposon-mediated depending on the bacterial species [8]. As in the case of TEM1, which hydrolyzes penicillins and first-generation cephalosporins [9], this variety has aided the dissemination of these enzymes. ESBLs produced by Enterobacteriaceae species have spread around the world since the introduction of novel medicines that target beta-lactamases [3-10]. Powder samples of Rhinacanthus nasutus plant leaves were taken and ethanol extract was prepared using soxhlet apparatus. The concentrated and dried extract was then subjected to phytochemical analysis. The bioactive components were identified by performing GCMS analysis, which showed the presence of eicosane as one of the bioactive component in the plant extract. Known data shows that eicosane showed potential antibacterial activity [11-12]. Therefore, it is of interest to document the molecular docking analysis of beta-lactamase from Salmonella species with eicosane.

## Methodology:

### Protein modelling and visualizations:

The protein sequence of beta-lactamase (Salmonella Sp.) was used for domain analysis using PFAM (https://pfam.xfam.org/). Then, the sequence was used for homology modelling server using Swiss Model (https://swissmodel.expasy.org/). The modelled protein 3D structure was validated using ProCheck server (https://saves.mbi.ucla.edu/) and viewed with the molecular visualization Software, Discovery Studio Software.

### 3D structure prediction for drug:

We used metronidazole, (CID: 4173) retrieved from NCBI –PubChem (https://pubchem.ncbi.nlm.nih.gov/) and data for the GC–MS instrument test compound, eicosane (CID: 8222) to perform molecular drug docking analysis. The 2D drug like compounds was converted into the 3D structure using Cheminformatics protocols.

### Molecular docking:

Molecular drug docking studies were performed using an automated molecular drug docking server, PatchDock (https://bioinfo3d.cs.tau.ac.il/PatchDock/). We docked the control drug (metronidazole) with beta-lactamase from Salmonella Sp and the test compound (eicosane) with beta-lactamase from Salmonella Species in order to compare the molecular binding affinities between the chemical molecules and the protein target.

## Results and Discussion:

The selected protein target was retrieved from NCBI database in FASTA format. The length of the Nucleotide sequence is 861 nt and corresponding amino acids sequence is 286 aa. The 3D structure of the target protein was developed using an automated homology modelling server named Swiss-Model. SWISS-MODEL server [13-16] converted the amino acid sequence of beta-lactamase from Salmonella Sp into 3D structure (Figure 1, 2, 3 & 4 - see PDF). The predicted structure was viewed using the molecular visualization tool, Discovery studio software. SWISS-MODEL [1-4] was used to analyse the molecular and structural details of beta-lactamase for docking. SWISS-MODEL is a server for automated comparative modelling of three-dimensional (3D) protein structures. Waterhouse et al. [13] computed models by the SWISS-MODEL server homology modelling pipeline which is based on ProMod3, an in-house comparative modelling engine based on Open Structure. The modelled 3D protein was comprehensively evaluated using the ProCheck server [6] for the assessment of Ramachandran Plot. The 3D structure of the mutated protein was validated using ProCheck server [17 - 18]. Figure 6(see PDF) shows the assessment of Ramachandran Plot which confirms that there is no error (90.5 %) in the modelled protein. Data shows that based on the molecular drug docking scores, the selected eicosane molecule is an efficient inhibitor of beta-lactamase (Salmonella Sp.) protein when compared to the control drug molecule metronidazole (Table 1 - see PDF).

Figure 7(see PDF) shows the 2D structure of metronidazole and Figure 8 represents the 3D structure of the metronidazole. Similarly, we show the 2D and 3D structure of eicosane in Figure 9 and 10, respectively. The conversion of 2D to 3D structure is one of the primary steps in drug docking procedure. We used Discovery Studio Software to perform automated 2D to 3D conversion for molecular docking using PatchDock. We ranked the remaining candidates as per a geometric shape complementarity score [19 – 20]. The PatchDock results of eicosane drug with beta-lactamase protein show an atomic contact energy value of -211.04 Kcal/mol (Figure 11 - see PDF). Whereas, that of the existing drug molecule, metronidazole with beta-lactamase protein is -121.25 Kcal/mol (Figure 9 - see PDF). Figure 11 & 12(see PDF) shows that binding between the target protein and the control drug. Figures 13 to 16(see PDF) show that eicosane is a potential inhibitor of beta-lactamase (Salmonella Sp.) protein. Interestingly, it was proved clinically that the domain region of beta-lactamase is found between 41 – 259 amino acids positions. Data show that eicosane directly binds within the range of the domain activity region of beta-lactamase (202-274 positions).

## Conclusions:

We document the molecular docking of beta-lactamase from Salmonella species with eicosane compared to metronidazole for further consideration.
